# Antioxidant lipoic acid ligand-shell gold nanoconjugates against oxidative stress caused by α-synuclein aggregates†

**DOI:** 10.1039/d0na00688b

**Published:** 2020-10-21

**Authors:** Maria Elena Piersimoni, Xiangyu Teng, Anthony E. G. Cass, Liming Ying

**Affiliations:** National Heart and Lung Institute, Imperial College London, Molecular Sciences Research Hub London W12 0BZ UK l.ying@imperial.ac.uk; Bio Nano Consulting London W1T 4TQ UK; Department of Chemistry, Imperial College London, Molecular Sciences Research Hub London W12 0BZ UK t.cass@imperial.ac.uk

## Abstract

Gold nanoparticles are becoming a promising platform for the delivery of drugs to treat neurodegenerative diseases. Parkinson's disease, associated with the aggregation of α-synuclein, is a condition that results in dysfunctional neuronal cells leading to their degeneration and death. Oxidative stress has been strongly implicated as a common feature in this process. The limited efficacy of the traditional therapies and the development of associated severe side effects present an unmet need for preventive and adjuvant therapies. The organosulfur compound lipoic acid, naturally located in the mitochondria, plays a powerful antioxidative role against oxidative stress. However, the efficacy is limited by its low physiological concentration, and the administration is affected by its short half-life and bioavailability due to hepatic degradation. Here we exploited the drug delivery potential of gold nanoparticles to assemble lipoic acid, and administered the system into SH-SY5Y cells, a cellular model commonly used to study Parkinson's disease. We tested the nanoconjugates of GNPs–LA, under an oxidative environment induced by gold nanoparticle/α-synuclein conjugates (GNPs–α-Syn). GNPs–LA were found to be biocompatible and capable of restoring the cell damage caused by high-level reactive oxygen species generated by excessive oxidative stress in the cellular environment. We conclude that GNPs–LA may serve as promising drug delivery vehicles conveying antioxidant molecules for the treatment of Parkinson's disease.

## Abstract 2

Lipoic acid–gold nanoconjugates (GNPs–LA) decrease GNPs–α-synuclein-induced oxidative stress on living cells, making them a promising antioxidant system for the treatment of Parkinson's disease.

## Graphical abstract

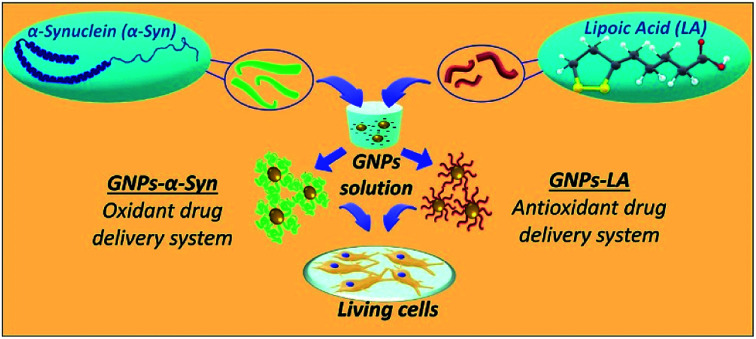

## Introduction

Parkinson's disease (PD) is the second most common neurodegenerative disease worldwide. It is characterized by the progressive loss of specific neuronal cells and its association with α-synuclein aggregation.^1,2^ The dysfunction and loss of neuronal cells that contribute to the disease pathogenesis might be the consequence of the oxidative stress generated by the overproduction of reactive oxygen species (ROS).^3–5^ The unregulated ROS react with many biomolecules resulting in the impairment of cellular functions and significant cellular damage such as lipid peroxidation, as well as oxidation of nucleobases in DNA and amino acid sidechains in proteins.^6–8^

Neuronal cells have been demonstrated to be highly sensitive to oxidative stress. Therefore, the roles of ROS in neurodegenerative diseases have been extensively studied to find effective antioxidant therapies able to prevent and decrease ROS generation.^9,10^

Currently, levodopa and dopamine agonists are considered the main treatments for PD, but these therapies have limited efficacy due to their severe side effects and low ability to penetrate the blood–brain barrier (BBB). Therefore, there is an unmet need to develop more effective therapies.^11–14^ The BBB is the highly selective membrane of the central nervous system (CNS) composed of endothelial cells which form tight junctions and separate blood from the extracellular fluid of the brain. The BBB is notably the main obstacle to therapeutic agents reaching the CNS. The application of nanomaterials to address medical questions is developing fast, thanks to the unique properties of such materials which remedy the intrinsic limitations of conventional drugs and enhance their therapeutic efficacy. The use of nanoparticles as drug delivery vehicles in the brain is of particular interest as they can pass through the BBB efficiently, thereby ensuring them to reach their targets in the treatment of neurodegenerative diseases.^15–17^ Gold nanoparticles (GNPs) have great potential as carriers for drug delivery based on their physicochemical properties, biocompatibility, stability, and ease of functionalization.^18–23^ The safety and effectiveness of GNPs in the treatment of PD in both cellular and animal disease models has already been proven and their utility is evidenced by the confirmed cellular activity of GNPs within the brains of PD patients, as reported in a Phase II clinical trial.^24–28^

**Fig. 1:**
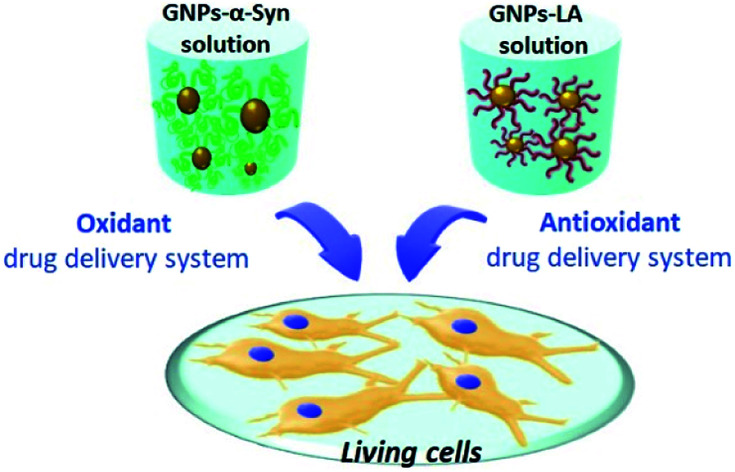
Schematic representation of the two nanodrug delivery systems, GNPs–α-Syn and GNPs–LA, tested on living cells. α-Synuclein and lipoic acid were tethered to the synthesized GNPs and, the respective oxidative and antioxidative systems obtained were tested on living SH-SY5Y cells.

The organosulfur compound lipoic acid (LA) has powerful antioxidant properties and the free molecule has already been employed as a less toxic therapeutic agent for neurodegenerative diseases.^29–31^ To overcome the limitations of the delivery of free lipoic acid due to its short half-life and low bioavailability,^32^ we sought to combine the drug delivery potential of GNPs with the compound to assess the ability of the self-assembled nanoconjugate system, termed GNPs–LA, to protect SH-SY5Y cells from ROS. To trigger oxidative stress in the SH-SY5Y cells, a widely used model for cellular studies of PD,^33^ an appropriate oxidative system based on GNPs and α-synuclein protein was prepared *via* electrostatic adsorption as shown in Fig. 1.

α-Synuclein (α-Syn) aggregation on 20 nm citrate capped GNPs enables the self-assembly of toxic α-synuclein aggregates^34^ on the GNP surface. These self-assembled aggregates on the shells of GNPs are responsible for increased oxidative stress in the cells, and consequently ROS levels due to both the physical disruption of the cell membrane and the direct binding with mitochondrial membrane proteins. Membrane disruption by the α-Syn aggregates leads to membrane permeabilization and increased lipid peroxidation, which, as one of the key factors of inducing death of neurons, is highly involved in the pathogenesis of PD.^35,36^

However, the mechanism of aggregation of α-Syn, a small intrinsically disordered protein of 140 amino acids, is complex and dependent on different environmental factors such as pH, temperature and contact with cell membrane components. It has been shown that the acceleration of α-Syn aggregation into the most toxic species of oligomers occurs through the enhancement of the primary nucleation step that precedes their transition to the fibril state whose accumulation then leads to the formation of Lewy body inclusions.^1,2^ Since GNPs were previously shown to act as an artificial chaperon system that was able to promote α-Syn aggregation by strongly influencing the primary nucleation step and the formation of the toxic oligomeric species^34^ that are responsible for triggering ROS in the cells, GNPs–α-Syn were selected as a viable oxidative system to study the antioxidant properties of GNPs–LA and explore their potential to treat the toxicity of the protein aggregation in PD diseases. Furthermore, GNPs as drug delivery vehicles are able to penetrate the cells efficiently^37^ thereby increasing the amount of α-Syn aggregates inside SH-SY5Y cells. Additionally, GNPs protect α-Syn from the proteolytic degradation,^37^ overcoming a key drawback of working with free proteins and peptides.

In this study, we synthetized and characterized two conjugates, GNPs–LA and GNPs–α-Syn, and evaluated their cytotoxicity to SH-SY5Y cells prior to the study of their uptake. We then examined the impacts of these two systems on ROS production and mitochondrial respiratory functions of SH-SY5Y cells and showed how the effects were correlated with both biophysical responses and structural changes in the microtubule cytoskeleton of the cells.

We demonstrate that GNPs–LA serve as antioxidant agents preventing the formation of ROS, restoring the mitochondrial respiratory functions, and protecting the SH-SY5Y cells from the structural alteration of the cell membrane associated with α-Syn aggregation.

## Results and discussion

### Nanoparticle synthesis and characterization

**Fig. 2:**
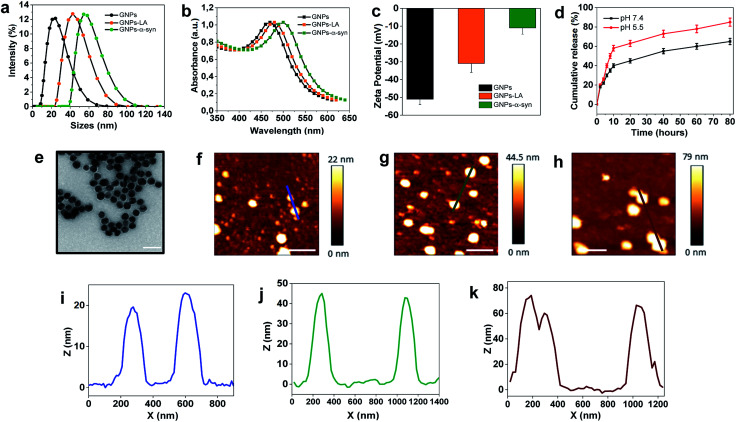
Characterization of GNPs, GNPs–LA and GNPs–α-Syn. (a) Hydrodynamic diameters of GNPs (black), GNPs–LA (orange) and GNPs–α-Syn (green) obtained by dynamic light scattering (DLS) in DMEM cell medium. (b) UV-Vis absorption spectra of GNPs (black), GNPs–LA (orange) and GNPs–α-Syn (green). (c) Zeta-potentials of GNPs, GNPs (black), GNPs–LA (orange) and GNPs–α-Syn (green) (mean ± SD, *n* = 3). (d) Release profiles of LA from GNPs–LA at pH 7.4 and pH 5.5. (e) Transmission electron microscopy image of GNPs, 500 000× magnification (scale bar 50 nm). (f–h) AFM topography of GNPs, GNPs–LA and GNPs–α-Syn (scale bar 200 nm). (i–k) Height profiles of GNPs, GNPs–LA and GNPs–α-Syn across the blue, green, red section lines, respectively.

The present study began with the synthesis and characterization of bare gold nanoparticles (GNPs)^38,39^ which were then conjugated with lipoic acid (GNPs–LA)^40,41^ and α-synuclein (GNPs–α-Syn).^34,42^ Bare negatively charged GNPs were synthesized according to the sodium citrate method. They were then tethered to LA and α-Syn respectively *via* the well-established thiol–gold bonding and electrostatic adsorption of α-Syn *via* its N-terminus, and purified in order to remove the free unbound LA and α-Syn in solution (see Assembly of GNPs–LA and GNPs–α-Syn in the Experimental section) and to avoid any interactions between the two free molecules when GNPs–LA and GNPs–α-Syn were mixed together. Dynamic light scattering (DLS) characterization showed an increase in the hydrodynamic diameters of ∼20 nm and ∼30 nm for GNPs–LA and GNPs–α-Syn, respectively, compared to that of unmodified GNPs with sizes of 20 nm, confirming successful conjugation (Fig. 2a). The polydispersity index values (PDI), indicators of the width of the nanoparticle distribution, were 0.18, 0.23, and 0.20 respectively (Table S1 in the ESI†). The conjugations of LA and α-Syn to GNPs were also confirmed by the shifts in UV-Vis absorption spectra (Fig. 2b) and changes in zeta potential (Fig. 2c). Due to the surface plasmon resonance (SPR) of the GNPs, their increase in the sizes can be determined through UV-Vis, in agreement with previous studies.^43,44^ The absorption peak at 480 nm of GNPs was attributed to the surface plasmon absorption of 20 nm spherical gold nanoparticles whereas, after the conjugation, a slight broadening of peaks was observed for both GNPs–LA (orange curve) and GNPs–α-Syn (green curve), in accordance with DLS results (Fig. 2b).

The conjugation of GNPs was also indirectly confirmed by the change in their corresponding Zeta Potential (ZP) (Fig. 2c). GNPs conjugated with LA were negatively charged as well as the citrate-stabilized GNPs, but they showed a less negative value of −35 ± 1 mV in comparison to that of citrate-stabilized GNPs (−53.6 ± 2 mV). The value for GNPs–α-Syn was −13 ± 5 mV, indicating that they were negatively charged at pH 7 due to their very acidic C-terminal region. These results suggested that direct interactions between the two differently conjugated GNPs are not favourable when the two systems were injected into the cells as a mixture. Although the aggregation of the nanoparticles does not depend solely on the charge, the magnitude of the ZP values obtained here was considered adequate to attain the stabilization of GNPs–LA and GNPs–α-Syn, taking into account of their sizes and the chemical structures of LA and α-Syn.^45^

The level of conjugation for LA was determined by UV-Vis absorption and found to be ∼20 LA molecules per nanoparticle. The level of conjugation was higher for α-Syn, around 100 α-Syn per nanoparticle. The release of LA from GNPs was examined at physiological and acidic pH (Fig. 2d). Cumulative release of LA was greater at both pH 5.5 than that at pH 7.4, with a percentage of 60% and 40%, respectively, after 10 h. Transmission Electron Microscopy (TEM) images showed that the bare GNPs had a similar spherical structure with the same morphology, and they were monodispersed exhibiting sizes of approximately 18 nm, in agreement with DLS measurements (Fig. 2e). Finally, atomic force microscopy analysis of the height of the three types of GNPs confirmed again that the height of bare GNPs (Fig. 2f) increased after the conjugation with LA (Fig. 2g) and α-Syn (Fig. 2h) and the sizes are consistent with those obtained by methods already mentioned. Bare GNPs had a height of ∼20 nm (Fig. 2i) whereas GNPs–LA (Fig. 2j) and GNPs–α-Syn (Fig. 2k) reached a height of ∼40 nm and ∼70 nm, respectively, in agreement with the DLS results.

### *In Vitro* cytotoxicity assay of GNPs–LA and GNPs–α-Syn

**Fig. 3:**
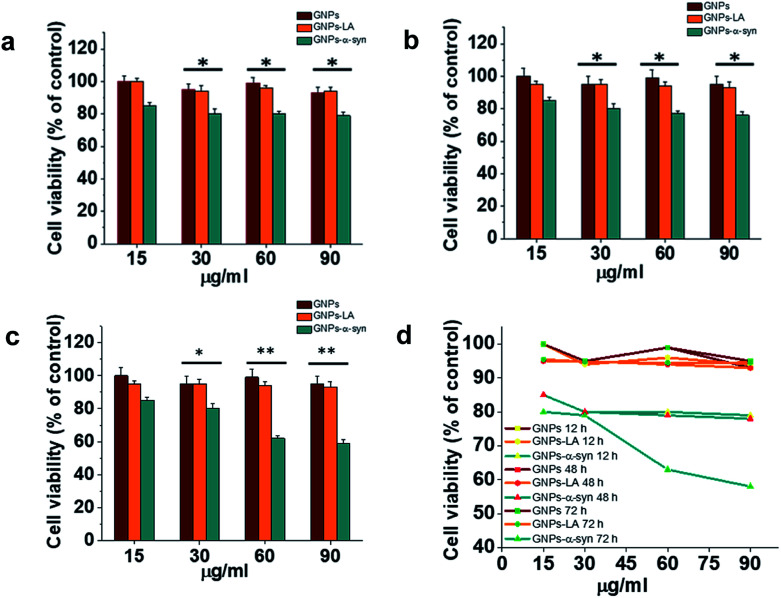
*In vitro* cytotoxicity of GNPs–LA and GNPs–α-Syn. SH-SY5Y neuroblastoma cell line was exposed to increasing concentration of GNPs (red), GNPs–LA (orange) and GNPs–α-Syn (green) for 12 h (a), 48 h (b) and 72 h (c). The cell viability, expressed in % in relation to the control with GNPs, was assessed by the MTT assay. Data are expressed as mean ± SEM, *n* = 3. *: *P* < 0.05; **: *P* < 0.01. (d) Dose–response curves of 15, 30, 60, and 90 μg ml^−1^ of GNPs, GNPs–LA and GNPs–α-Syn on SH-SY5Y cell viability. Cells were seeded in a confluent monolayer in a 96 well plate and exposed to GNPs, GNPs–LA and GNPs–α-Syn for 12 h, 48 h and 72 h.

Cytotoxicity evaluations of GNPs, GNPs–LA and GNPs–α-Syn on SH-SY5Y neuroblastoma cells were performed using the MTT assay. A dose–response treatment was conducted by placing a confluent monolayer of cells in a 96 well plate and incubating them with 15, 30, 60, and 90 μg ml^−1^ of nanoparticles for 12 h (Fig. 3a), 48 h (Fig. 3b) and 72 h (Fig. 3c). The cells were then washed twice and incubated with a solution of 3-(4,5-dimethylthiazol-2-yl)-2,5-diphenyltetrazolium bromide (MTT) at 37 °C for 45 minutes until the formation of formazan crystals. The samples were then analysed spectrophotometrically by monitoring the absorption at 570 nm.

Bare GNPs and GNPs–LA were not toxic at any of the doses investigated, consistent with previous reports,^23,41,44,46^ while a dose and incubation time dependent decrease in cell viability was observed when the cells were exposed to GNPs–α-Syn. In general, no significant reduction was seen after 12 h, 48 h, and 72 h at four doses of 15, 30, 60, and 90 μg ml^−1^ GNPs–LA. Instead, there was reduction in cell viability with GNPs–α-Syn, 15% in the case of 15 μg ml^−1^ and 20% in the case of 30, 60, and 90 μg ml^−1^ compared to bare GNPs for 12 h and 48 h incubation. After 72 h and at concentrations of 15 and 30 μg ml^−1^, GNPs–α-Syn induced 20% reduction in cell viability while ∼40% at concentrations of 60 and 90 μg ml^−1^ compared to the same doses of both GNPs and GNPs–LA (Fig. 3c and d). To elucidate the cytotoxic effect of α-Syn in the form of aggregates when it is electrostatically tied with GNPs, the effect of monomeric α-Syn at different doses was investigated (Fig. S2 in the ESI†). Equimolar free monomeric α-Syn was less cytotoxic than GNPs–α-Syn at the doses studied. At 72 h and 60 μg ml^−1^ GNPs–α-Syn, cell viability was decreased by ∼40%, whereas α-Syn at the same concentration (equivalent to 0.6 μM) caused lower viability loss, at 15%. The same was observed in the case of increased doses at 90 μg ml^−1^ GNPs–α-Syn and equivalent 0.9 μM free α-Syn where the latter showed again cell viability loss of 20%, less than that of ∼40% caused by GNPs–α-Syn. The increased cytotoxicity of GNPs–α-Syn compared to that of free α-Syn can be attributed to the effect of the GNPs on the aggregation of the α-Syn and consequently the disruption of the cell membrane, as previously suggested,^2,47,48^ but it can also be a result of a rise of the amount of GNPs–α-Syn internalized in the cells due to the higher uptake of the nanoparticles.

### Cellular uptake of GNPs–LA and GNPs–α-Syn

**Fig. 4:**
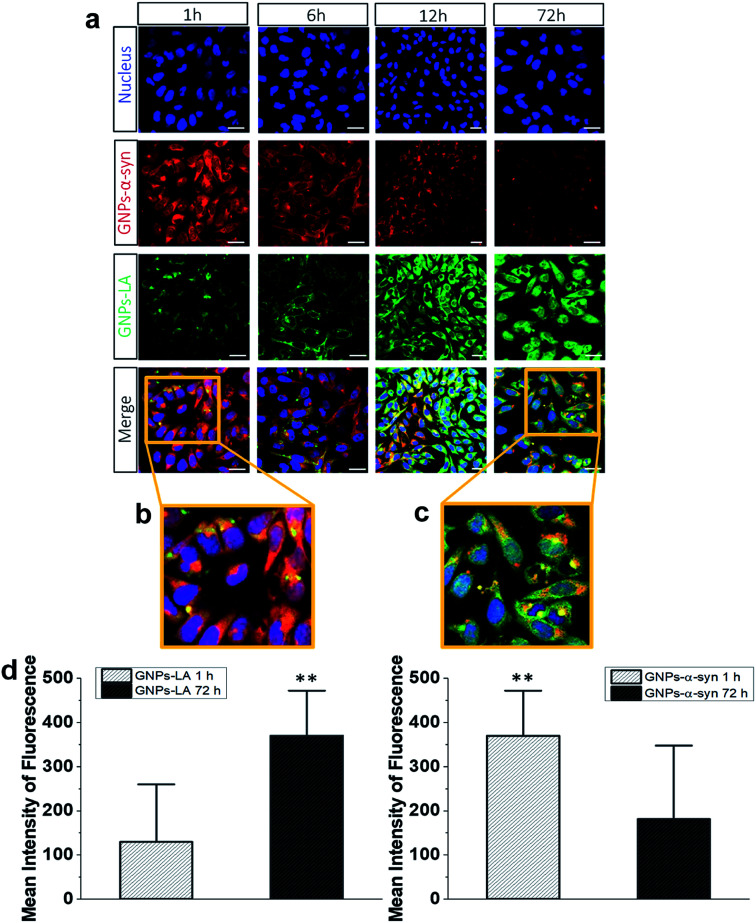
Intracellular uptake and distribution of GNPs–α-Syn (red, AlexaFluor 647) and GNPs–LA (green). (a) Live SH-SY5Y cells were exposed to GNPs–LA and GNPs–α-Syn 1 h, 6 h, 12 h and 72 h prior to imaging by confocal microscopy. The nucleus is visualized in blue. Scale bar = 10 μm. (b) Zoomed merged images of intracellular uptake of GNPs–LA (green) and GNPs–α-Syn (red) after 1 h. (c) Zoomed merged images of intracellular uptake of GNPs–LA (green) and GNPs–α-Syn (red) after 72 h incubation; scale bar = 10 μm. (d) The mean fluorescence intensity of GNPs–LA (left) and GNPs–α-Syn (right) by confocal microscopy in SH-SY5Y cells after 1 h and 72 h exposure of both GNPs–LA and GNPs–α-Syn. Data are given as mean ± SEM; *n* = 50 cells. **: *p* < 0.01.

To understand the intracellular fate of both GNPs–LA and GNPs–α-Syn, the uptake of the particles by SH-SY5Y living cells was investigated using confocal microscopy. After observing the highest viability loss of 40% for 60 μg ml^−1^ GNPs–α-Syn in the cytotoxicity studies, the dose of 60 μg ml^−1^ was selected for the cellular uptake studies. A mixture of GNPs–LA and GNPs–α-Syn was injected to 8-well chamber slides where the cells were plated at a density of 2 × 10^5^ cells per well. The uptake was studied after 1 h, 6 h, 12 h and 72 h of incubation and the cells were washed three times with Dulbecco's Phosphate-Buffered Saline (DPBS) prior to performing fluorescence imaging to avoid the sedimentation of the particles. The localization of GNPs–LA visualized in green due to luminescence from the nanoparticles and red fluorescence from dye labelled GNPs–α-Syn (see α-Synuclein expression and labelling in the Experimental section) can be detected in the cells demonstrating the internalization of GNPs–LA in the cytoplasm and even close to the nucleus, and both the cytoplasm and nucleus uptake of GNPs–α-Syn within cells (Fig. 4a). At 1 h of exposure of particles, a higher uptake was seen for the GNPs–α-Syn (Fig. 4b). After 6 h, the uptakes of both GNPs–LA and GNPs–α-Syn were shown to be similar, illustrated by a balanced fluorescence signal from these two types of nanoparticles. When the cells were exposed to the nanoparticles for a prolonged time (12 h and 72 h), an increase in the cellular internalization of GNPs–LA was observed in comparison to GNPs–α-Syn at the same injected doses (Fig. 4a–c). Although the internalization routes of nanoparticles may be influenced by their sizes, shapes and surface coatings, the presence of LA on GNPs–LA may interfere with the accumulation of α-synuclein in a longer time duration. The acidic environment of the lysosomes where GNPs–LA were accumulated can promote the Au–S bond breakage and consequently a major release of LA from the conjugates after 12 h. This continuous release of LA from GNPs within the cells results in a significant accumulation of LA which may influence the accumulation of α-synuclein in the intracellular compartment, as it has been shown previously.^49,50^ Since the accumulation of α-synuclein occurs on plasma membranes due to its association with the phospholipids with good affinity,^51^ it may be postulated that the α-synuclein shell of GNPs decreases the internalization of GNPs–α-Syn, thereby promoting their binding to the cell membrane and accumulating there from 6 h exposure and thereafter. The quantification of the mean fluorescence intensities of GNPs–LA and GNPs–α-Syn confirmed the higher internalization of GNPs–α-Syn at 1 h exposure and then it decreases (72 h), whereas an opposite trend was observed for GNPs–LA (Fig. 4d).

To directly compare the intracellular uptake and distribution of the antioxidant system GNPs–LA with free LA, cellular uptake studies were also carried out after incubation of the cells with free LA for 24 h and 72 h. Interestingly, the internalization of free LA was much lower than that of GNPs–LA as early as 24 h of exposure to the cells, showing an ∼3 fold-decrease in the mean fluorescence intensity of free LA compared to that of GNPs–LA after 72 h (Fig. S3 in the ESI†). These results indicate that GNPs are required as carriers for the efficient uptake and internalization of LA by the cells. Furthermore, GNPs–LA exhibit increased solubility compared to both free LA and the bare GNPs, leading to higher accumulation of them in the cells in shorter times, in good agreement with previous studies.^52^ This property is known to withstand the hepatic clearance and prolong the circulation half-life of the drug delivery system *in vivo*, enabling cumulative delivery of drugs to the target cells.^53^ The internalization mechanisms of both GNPs–LA and GNPs–α-Syn are not known, so future investigation into this aspect is desirable. However, the proximity of the nanoparticles to the cytoplasm and lysosomes suggests that GNPs–LA and GNPs–α-Syn might employ the endocytic pathway to enter the cells. This mechanism is common for GNPs of similar sizes.^19,21,23^

### Mitochondrial oxygen consumption rate (OCR) measurement

Having investigated the uptake of GNPs–LA and GNPs–α-Syn and found them internalized into the cells, mitochondrial respiratory functions of the cells before and after the nanoparticle exposure were analyzed using the Seahorse XFe96 analyzer.

To evaluate if GNPs–LA and GNPs–α-Syn exposure to cells affected the mitochondrial respiratory function, mitochondrial oxygen consumption rates (OCR) of SH-SY5Y live cells were investigated.

The cells were seeded in 24-well plates and exposed for 24 h and 72 h to GNPs (60 μg ml^−1^), GNPs–LA 1/2 (60 μg ml^−1^ and 30 μg ml^−1^), GNPs–α-Syn (60 μg ml^−1^), GNPs–LA 1/2 (60 μg ml^−1^ and 30 μg ml^−1^)/GNPs–α-Syn 1/2 (60 μg ml^−1^ and 90 μg ml^−1^), and to 6-hydroxydopamine (6-OHDA) (35 mM). Before the analysis, the cells were washed with Dulbecco's Phosphate-Buffered Saline (DPBS) and Dulbecco's Modified Eagle's Medium (DMEM) was added to the cells that were then incubated at 37 °C in a humidified atmosphere of 5% CO_2_ and 95% air for 1 h. After the measurement of the baseline (three baseline measurements were recorded), the three different modulators of mitochondrial respiration were added sequentially. 1 μM oligomycin, an inhibitor of ATP synthase, 0.0125 μM carbonyl cyanide-4 (trifluoromethoxy) phenylhydrazone (FCCP), an uncoupling agent that disrupts the mitochondrial membrane potential, and 1 μM antimycin/rotenone, Complex I and III inhibitors were injected in succession through the ports A, B, and C of the Seahorse XFe96 analyzer, respectively.

After both 24 h and 72 h, the exposure of the cells to GNPs–LA (60 μg ml^−1^) induced a significant increase of maximal respiration (33.2% and 43.4% respectively), ATP production (50% and 77% respectively) and spare respiratory capacity (65.6% and 80% respectively) (Fig. S4A and B in the ESI†) compared to that of the cells treated with GNPs–α-Syn (60 μg ml^−1^) (Fig. S4C and D in the ESI†).

As a negative control to validate the toxic effect of GNPs–α-Syn on SH-SY5Y cells, the cells were also treated with 6-OHDA, a neurotoxin that forms free radicals and inhibits the Complexes I and IV of the mitochondrial respiratory chain.^54^ The decreases in the level of maximal respiration, ATP production and spare respiratory capacity after GNPs–α-Syn exposure were comparable to those when the cells were exposed to 6-OHDA for both 24 h and 72 h (Fig. S4C and D in the ESI†), suggesting that GNPs–α-Syn, accumulated presumably in the mitochondria, can promote the alteration of their respiratory function and induce the formation of ROS, which lead to oxidative stress. The negative control of cells treated with neurotoxin 6-OHDA allowed us to ensure that the mitochondrial toxicity triggered by GNPs–α-Syn is comparable to that of 6-OHDA, a widely used reagent to reproduce several cellular processes identified in PD.

As been previously shown, the interaction of α-synuclein with mitochondria also impairs mitochondrial function through the inhibition of Complex I, enhancing the ROS level thereby resulting in cell damage and reduction of cell viability.^32,54–56^ After 72 h exposure to GNPs–α-Syn, ATP production and spare respiratory capacity were significantly lower than those under exposure to 30 μg ml^−1^ and 60 μg ml^−1^ GNPs–LA, and the GNPS–LA (30 μg ml^−1^)/GNPs–α-Syn (30 μg ml^−1^) mixture (Fig. S4D in the ESI†). In the case of 30 μg ml^−1^ GNPs–LA the levels of maximal respiration, ATP production and spare respiratory capacity rose by 37%, 16% and 11% respectively compared to the levels after exposure to the GNPs–LA (15 μg ml^−1^)/GNPs–α-Syn (45 μg ml^−1^) mixture (Fig. S4D in the ESI†). Instead, when the cells were exposed to the GNPS–LA (30 μg ml^−1^)/GNPs–α-Syn (30 μg ml^−1^) mixture, the level of basal respiration and maximal respiration increased by 55 and 100 pmol O_2_ per min compared to when the cells were subjected to the exposure of GNPs–α-Syn alone.

These experiments clearly demonstrated the antioxidant effect of GNPs–LA, consistent with literature studies^55,57,58^ where lipoic acid was found to act as both a powerful antioxidant and enzyme bound-cofactor for mitochondrial 2-ketoacid dehydrogenase which contributes to the production of ATP in the cells. Moreover, this restorative effect could also arise from one of the important physiological functions of lipoic acid-activating the mitochondrial medium-chain acyl CoA synthetase (ACSM1), which uses both the exogenous (*S*) and (*R*)-enantiomers of LA to activate GTP to produce the endogenous lipoic acid in a positive feedback.^32,55,56^

The analysis of the dependence of the OCR value on various types of nanoparticles showed that 72 h GNPs–α-Syn exposure led to a higher reduction of basal respiration compared to that under the exposure of 24 h, whereas exposure to GNPs–LA (60 μg ml^−1^) yielded an increase in the basal OCR, compared to all other cases (Fig. S4E and F in the ESI†). OCR levels after exposure for 72 h to 60 μg per ml of 1 : 1 GNPs–LA/GNPs–α-Syn mixture did not significantly change in comparison to the exposure after 24 h. However, the presence of GNPs–LA together with GNPs–α-Syn significantly affected the rise of basal respiration, maximal respiration, ATP production and spare respiratory capacity compared to the values when the cells were exposed to GNPs–α-Syn alone.

The beneficial effects we have seen when the cells were exposed to both 30 μg ml^−1^ and 60 μg ml^−1^ GNPs–LA did not show up when the treatment was with the bare GNPs, where the level of maximal respiration and spare respiratory capacity stayed the same at 80 and 40 pmol O_2_ per min respectively in either 24 h or 72 h experiments. These values are much lower than those observed for 30 μg ml^−1^ and 60 μg ml^−1^ GNPs–LA in 72 h experiments (160 and 70 pmol O_2_ per min and 125 and 50 pmol O_2_ per min, respectively) (Fig. S4E and F in the ESI†). These results suggested that although the bare GNPs have been shown to be nontoxic towards the cells based on our current cytotoxicity studies and the literature,^20,23^ they do not possess antioxidant properties and do not increase the cell's ability to produce more ATP. Instead they decrease the cell's ability to cope with oxidative stress. GNPs–LA, on the other hand, show the opposite effects.

However, these results corroborate well with those observed in the cell viability assay, indicating that GNPs and GNPs–LA are not toxic at the doses investigated and that GNPs–LA possess a strong alleviating effect on the cell toxicity. GNPs–LA have enhanced the maximal respiration and spare respiratory capacity of mitochondria, where the energy reserve increases the cell viability when the demand for energy in the cells is high under oxidative stress conditions.^58,59^

Taking this observation into consideration, all the later experiments were carried out using the 60 μg ml^−1^ GNPs–LA concentration and the GNPs–LA (30 μg ml^−1^)/GNPs–α-Syn (30 μg ml^−1^) mixture that showed the most promising results.

### Lipid peroxidation measurements

**Fig. 5:**
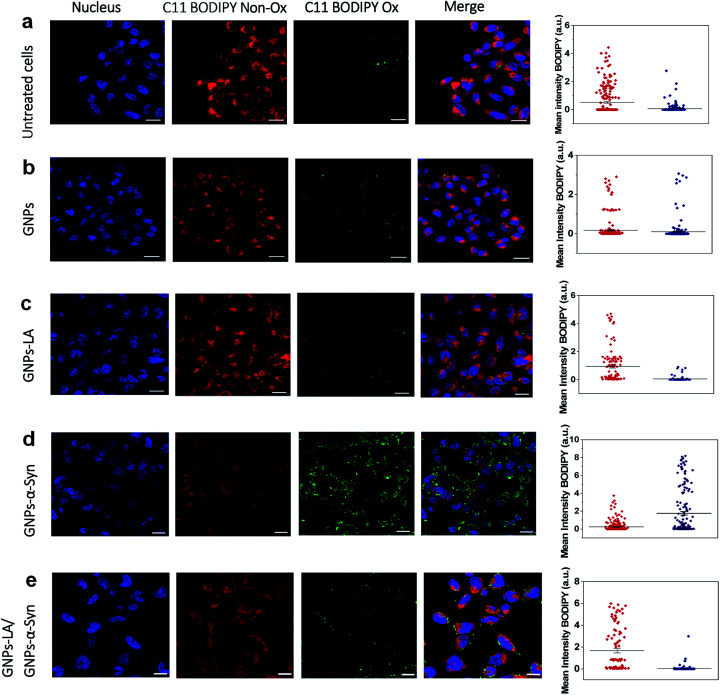
Lipid peroxidation measurements using C11 BODIPY in untreated and treated SH-SY5Y live cells with the nanoparticles (60 μg ml^−1^). (a) Lipid peroxidation levels in untreated SH-SY5Y live cells and (b) after the exposure to GNPs. (c) Lipid peroxidation measurement after SH-SY5Y live cells were exposed to GNPs–LA. (d) Lipid peroxidation measurement after the exposure to GNPs–α-Syn and (e) GNPs–LA/GNPs–α-Syn together prior to imaging by confocal microscopy. The nucleus is visualized in blue. Scale bar = 10 μm. The mean fluorescence intensity of C11 BODIPY non-oxidized (red) and the shift of the emission peak from non-oxidized to oxidized (green) for each experiment are shown on the right panel and data are given as mean ± SEM; *n* = 70 cells.

After confirming the effect of GNPs–LA and GNPs–α-Syn and knowing that there is a strong correlation between the mitochondrial energy imbalance and the ROS formation, we next explored the effect of the nanoparticles on lipid peroxidation in SH-SY5Y living cells and in real-time by confocal microscopy. The use of the specific C11-BODIPY lipid peroxidation sensor enabled us to detect the presence of ROS in the cell membranes, and in particular, to quantify the fluorescence shift from 590 nm (red) to 510 nm (green) due to the oxidation of the probe by increasing the level of the peroxyl radical, one of the key reactive oxygen species involved in the induction of PD.^60^ SH-SY5Y cells were plated on 8-well chamber slides at a density of 2 × 10^5^ cells per well and incubated overnight. We conducted the analysis in untreated SH-SY5Y live cells (Fig. 5a) and after cell exposure to 60 μg ml^−1^ GNPs (Fig. 5b), 60 μg ml^−1^ GNPs–LA (Fig. 5c), 60 μg ml^−1^ GNPs–α-Syn (Fig. 5d), and the GNPs–LA (30 μg ml^−1^)/GNPs–α-Syn (30 μg ml^−1^) mixture (Fig. 5e). At the same dose investigated, there were no significant differences in the shifts in fluorescence between the cells treated with GNPs and GNPs–LA, as well as the control where the cells were untreated (Fig. 5a), implying that the treatments did not induce noticeable lipid peroxidation.

However, the shift trend between the Non-Ox and Ox forms of the sensor was more similar when the cells were untreated (control) and treated with GNPs–LA, as shown in the data plots where the mean intensities of BODIPY Ox have decreased by ∼2 fold compared to the BODIPY Non Ox for both untreated (Fig. 5a) and GNPs–LA treated cells (Fig. 5c). This confirms that the presence of LA on GNPs shells plays a beneficial protective role in lipid peroxidation, consistent with the study reported by Abdelhalim *et al.*^61^ where the use of LA conjugated GNPs prevented some of the toxic effects caused by the clearance of bare GNPs *in vivo*, including lipid peroxidation, nephrotoxicity and inflammatory kidney damage. When the cells were exposed to 60 μg ml^−1^ GNPs–α-Syn (Fig. 5d), the level of the oxidized probe increased by about two-fold, indicating that GNPs–α-Syn induced significant lipid peroxidation which is a signal of the elevation of the ROS level in the cells caused by the misfolding and aggregation of α-synuclein.^62^ When the lipoic acid–gold nanoconjugate system was injected together with GNPs–α-Syn, the amount of oxidized probes was significantly reduced, as represented by the green fluorescence image, as shown in Fig. 5e and the corresponding data plot. The magnitude of the reduction of the oxidized probe is greater for GNPs–LA than in the case of the injection of the equivalent dose of free LA (Fig. S5 in the ESI†), further confirming the efficacy of the GNPs–LA system and its higher internalization into the cells, as already demonstrated by our cellular uptake studies (Fig. 4 and S3 in the ESI†). Similar to that, the lipid peroxidation control measurements carried out with free α-Syn (Section 2.5 of the ESI†) have confirmed that GNPs–α-Syn have increased the lipid peroxidation level by about ∼6 fold compared to free α-Syn (Fig. S5 in the ESI†), further proving the oxidative ability of GNPs–α-Syn.

In conclusion, while the α-synuclein aggregates on the surface of GNPs strongly enhanced the oxidation process in the cells and damaged the lipid membranes, GNPs–LA did have efficacy in reducing lipid peroxidation. These results provide strong evidence that GNPs–LA well preserve the antioxidant system of the SH-SY5Y living cells compared to LA, confirming the biocompatibility of the GNPs–LA system and its efficacy against the oxidative stress in PD.

### Nano-mechanical studies of SH-SY5Y living cells upon nanoparticle exposure

After investigating the antioxidative capacity of GNPs–LA, we next examined the cell membrane response of SH-SY5Y cells before and after the nanoparticle exposure using a Bio AFM system which allows for label-free and real-time nano-mechanical studies.

**Fig. 6:**
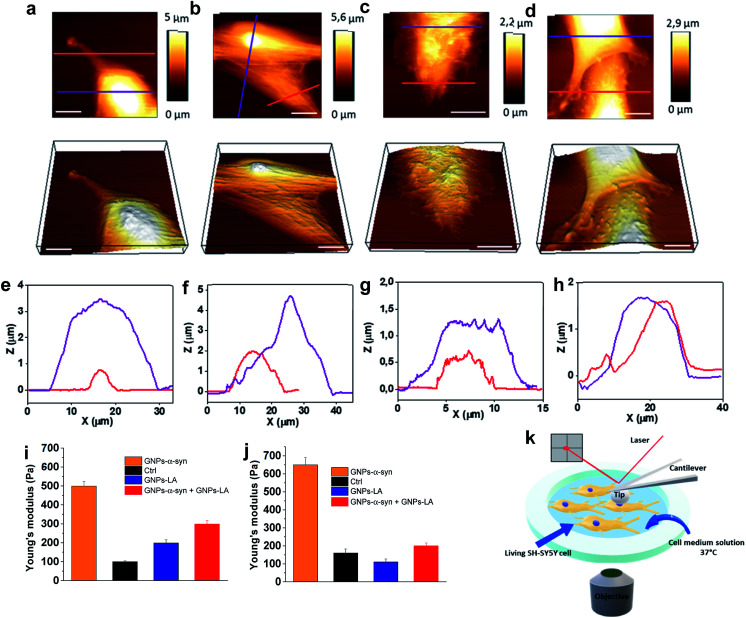
Nano-mechanical response of SH-SY5Y living cells upon nanoparticle exposure (60 μg ml^−1^). (a) Representative AFM images of untreated SH-SY5Y live cells and (b) SH-SY5Y live cells exposed to 60 μg ml^−1^ GNPs–LA, (c) GNPs–α-Syn and (d) the GNPs–LA/GNPs–α-Syn mixture. Corresponding 3D topographic images are shown in the lower panels. (e) Height profile of untreated SH-SY5Y live cells and (f) SH-SY5Y live cells treated with GNPs–LA, (g) GNPs–α-Syn and (h) the GNPs–LA/GNPs–α-Syn mixture across the lines in the topographic AFM images. Scale bar = 10 μm. (i and j) Young's modulus mean values obtained from AFM measurements on SH-SY5Y living cells after the exposure to GNPs–LA, GNPs–α-Syn and the GNPs–LA/GNPs–α-Syn mixture for 24 h and 72 h. Error bars represent the SEM of the mean for each condition, *n* = 30 cells. (k) Schematic of the setup for the nano-mechanical studies.

The measurements were performed on untreated cells (Fig. 6a) and those treated with the nanoparticles under physiological conditions at 37 °C. Prior to the measurements, the cells were exposed to 60 μg ml^−1^ of GNPs–LA, 60 μg ml^−1^ GNPs–α-Syn and the GNPs–LA (30 μg ml^−1^)/GNPs–α-Syn mixture (30 μg ml^−1^) (Fig. 6b–d) for 24 h and 72 h. To allow a uniform distribution of the force applied to the cells and to make accurate measurements, the elasticities of the untreated and treated cells were probed on top of the nucleus on single cells (see the schematic in Fig. 6k). This approach together with the spherical shape of the indenter ensures a homogeneous distribution of the force applied to the cell body, minimizing the risk of the membrane breakage and/or damage.^63,64^ Force indentation curves were recorded at a set point of 1.5 nN with a constant ramping speed of 5 μm s^−1^. They were then fitted with the JPK data processing software using the Hertz model for the spherical indenter.^63^ The structure and the biophysical properties of the cells play a crucial role in a variety of cell functions such as motility, signal transduction, cytoskeleton structures and consequently bioenergetic function.^65^ Since the external perturbation due to the force applied onto the cells could trigger diverse biochemical changes, we probed ∼30 cells in each experiment to account for this variability. The structural changes of treated cells were first investigated by imaging the cells before and after 72 h treatment with GNPs–LA and the GNPs–LA/GNPs–α-Syn mixture. No significant structural differences were observed for the untreated cells and for the cells exposed to GNPs–LA. In fact, the plasma membranes were not disrupted by lipid peroxidation as demonstrated in the lipid peroxidation experiment, and their fibroblastic-like morphology as well as the biophysical properties were maintained as indicated by their elongated shape. The height of both the untreated cells and those treated with GNPs–LA was around 1–2 μm on the edge areas, reaching 5–6 μm on the central areas atop the nuclei, as shown in the cross-line height profiles plotted in Fig. 6e and f. When we imaged the cells 72 h post GNPs–α-Syn exposure, pore-like defects were observed, suggesting the disruption of the cell membrane (Fig. 6c). In addition, shrinking of the cells with a reduction in the cell heights and an increase in their roughness were observed (Fig. 6g), leading to the assumption that an instability of microtubules in the cytoskeleton of the cells might arise due to GNPs–α-Syn exposure.

Lipid peroxidation disrupts the integrity of the membrane and its functions, hence inducing cell death, as demonstrated by Agmon *et al.*,^66^ where they simulated how lipid hydroperoxide influences membrane properties. This correlation is consistent with the present finding in which the exposure of GNPs–α-Syn triggered lipid peroxidation in the cells, resulting in cell membrane disruption, whereas the exposure of GNPs–LA, an antioxidant system, did not. Our result is also consistent with the study of Sinha *et al.*^67^ where the nano-structural alterations of red blood cells upon chemically induced oxidative stress using different oxidizing agents, including hydrogen peroxide and cumene hydroperoxide, were evaluated. In addition, it is notable that the oxidative damage triggered by the GNPs–α-Syn system induced critical changes to the cytoskeleton structure of the SH-SY5Y cell membrane (Fig. 6c), implying a possible reorganization of the membrane components.

To probe the influence of the antioxidant GNPs–LA on the structure of the SH-SY5Y cell membrane when the cells were exposed to the oxidative damage, a mixture of GNPs–LA/GNPs–α-Syn was also injected. Similar to the case of cells exposed to GNPs–α-Syn, the height of the cells was found to be lower than those of both the untreated cells and cells treated with GNPs–LA, which reached barely 2 μm on the central cell area. However, the membrane disruption and the defects were no longer observed, and the structure did not seem to be altered (Fig. 6d and h). This restoration of the cell membrane to the normal state when the cells were exposed to GNPs–α-Syn and GNPs–LA together was also reinforced by Young's modulus values calculated for the four different experimental conditions. Using the same probing conditions, the Young's modulus mean values were not significantly different between the untreated cells and the cells treated with GNPs–LA, ranging from 200 to 250 Pa after both 24 h and 72 h. When the cells were exposed to GNPs–α-Syn alone, the Young's Modulus values rose to 500 and 650 Pa, respectively (Fig. 6i and j). Thus, the exposure of GNPs–α-Syn to the cells resulted in an increase in the stiffness of the cell membrane due to an increase in its rigidity that was restored to physiological conditions by the GNPs–LA treatment.

The increase in membrane stiffness induced by oxidant treatments, including H_2_O_2_ and OH, has been observed in previous studies.^67,68^ Yusupov *et al.*^68^ attributed this phenomenon to the thickened lipid bilayer. On the other hand, Sinha and co-workers^67^ found the shrinking of the cytoskeleton and consequently a reduced membrane deformability and height, as evidenced from their AFM studies. Our result seems to support the latter as a decrease in cell height was observed. The cell membrane is one of the preferential targets of ROS which cause lipid peroxidation because the chains of polyunsaturated phospholipids are particularly vulnerable to the attack. Lipid peroxidation disturbs the bilayer structure by modifying the membrane fluidity and contributes to the membrane damage. There is a strong correlation between lipid peroxidation and the reduction in membrane fluidity, implying that oxidative stress induces membrane rigidity due to the modification of the physicochemical properties of the lipid bilayer, as previously suggested.^69^ Also, the cytoskeleton elements such as microfilaments and microtubules, attached to the cell membrane, are crucial for mitochondrial morphology, organization, and respiratory functions.^70^ The results of the lipid peroxidation assay and mitochondrial oxygen consumption rate measurements demonstrated that GNPs–LA can prevent the damage induced by the ROS formation, in good agreement with the scenario discussed above. Furthermore, since no membrane disruption and alteration were observed, the biocompatibility of GNPs–LA was also confirmed. Although previous studies have shown the efficacy of GNPs–LA in cancer treatment,^51^ to the best of our knowledge, this is the first time that both the nano-mechanical response of the cytoskeleton of living cells and the change in membrane stiffness of these cells were probed when the antioxidant GNPs–LA system was employed as an anti-PD therapy.

### Confocal imaging of the microtubule cytoskeleton in SH-SY5Y living cells

**Fig. 7:**
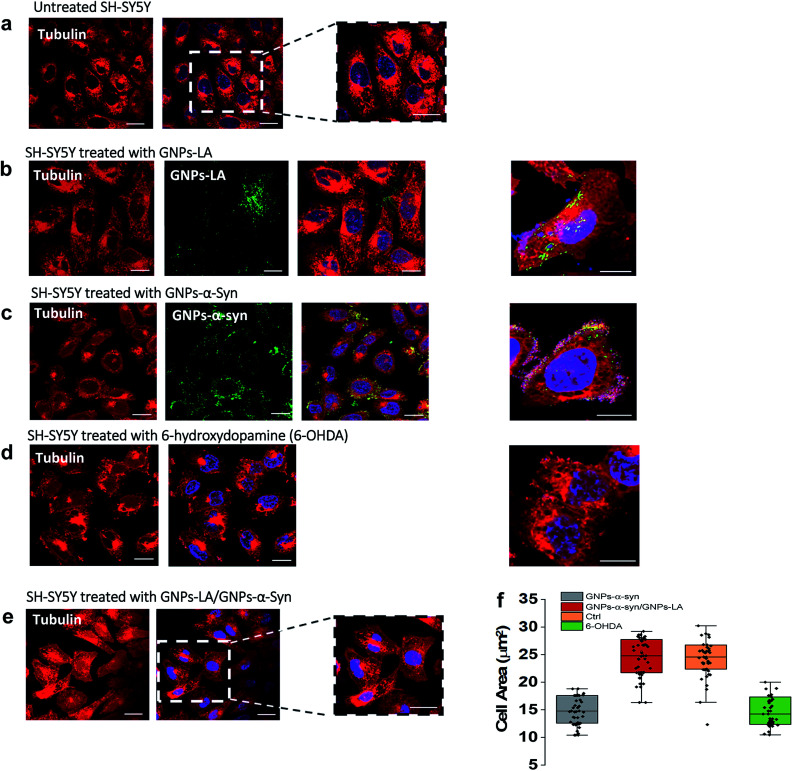
Changes in the microtubule cytoskeleton induced by 72 h exposure to 60 μg ml^−1^ nanoparticles investigated by confocal imaging using SiR-tubulin protein. Scale bar = 10 μm. (a) Untreated SH-SY5Y. (b), (c) and (d) are SH-SY5Y treated with GNPs–LA, GNPs–α-Syn and negative control 6-hydroxydopamine, 6-OHDA, respectively. (e) Microtubule structure of SH-SY5Y exposed to the mixture GNPs–LA/GNPs–α-Syn prior to imaging by confocal microscopy. GNPs–LA and GNPs–α-Syn are visualized in green. (f) Cell body area analysis of SH-SY5Y cells under different treatment conditions. Error bars represent the SEM of the mean for each condition, *n* = 70 cells.

Finally, we explored the effect of 60 μg ml^−1^ GNPs–LA, 60 μg ml^−1^ GNPs–α-Syn and the GNPs–LA (30 μg ml^−1^)/GNPs–α-Syn (30 μg ml^−1^) mixture on the microtubule assembly of SH-SY5Y living cells after 72 h exposure of nanoparticles by means of confocal imaging. In order to determine the effect of GNPs–α-Syn and GNPs–LA with the microtubule cytoskeleton structure, the untreated cells (Fig. 7a) and cells exposed to GNPs–LA (Fig. 7b), GNPs–α-Syn (Fig. 7c), and the mixture of GNPs–LA/GNPs–α-Syn (Fig. 7e) were incubated with the microtubule protein SiR-tubulin.

As a negative control, the neurotoxin 6-OHDA was used as a drug to increase ROS production (Fig. 7d). This strategy, as previously explained, enabled us to compare the effect of GNPs–α-Syn on the microtubule structure with respect to the negative control where SH-SY5Y cells underwent drug treatment which elicits ROS production. When cells were treated with GNPs–LA (Fig. 7b), a cluster of GNPs–LA appeared to localize throughout the cytoplasm and in the perinuclear and nuclear structures. No apparent changes were found in the morphology and area of the cells compared to the untreated cells (Fig. 7a). However, after 72 h exposure to the GNPs–α-Syn, there was a significant change in the morphology of the cells compared to both the untreated cells and the cells exposed to GNPs–LA alone. Cells treated with GNPs–α-Syn (Fig. 7c) instead had a similar morphology to the negative control, the 6-OHDA treated cells (Fig. 7d), further confirming the oxidative effect of GNPs–α-Syn, as observed in the lipid peroxidation assay. In addition, akin to the cells treated with 6-OHDA again, cells exposed to GNPs–α-Syn were more spherical than elongated with a lower cell body size of ∼10–15 μm, compared to both the untreated and treated cells with GNPs–LA, as shown in Fig. 7f. Moreover, the cell membrane became incomplete, particularly in the areas where the fluorescence of GNPs–α-Syn was localized, indicating that GNPs–α-Syn may disrupt the microtubule structure and consequently provoke the unhealthy appearance of the cell, consistent with the results of cytotoxicity studies. When the cells were exposed to GNPs–α-Syn and GNPs–LA together, a protective effect on the microtubule structure was observed as the cell morphology reassumed the elongated shape of the untreated cells, suggesting that the tubulin was not compromised. Similarly, the cell body reacquired the regular size of ∼20–30 μm, which is the same as that of the untreated cells (Fig. 7f).

The neuroprotective capacity of LA in the neurodegeneration has been reported recently, which demonstrated that therapeutic treatment with LA preserved the survival of the retinal ganglion cells.^71^ Moreover, Melli *et al.*^72^ showed that LA exerts neuroprotective effects by preventing the mitochondrial dysfunction *via* the expression of the mitochondrial iron chaperone or iron storage protein, frataxin. Although more studies would be needed to shed light into the precise action mechanism of GNPs–LA, our work is in agreement with these studies and further provided the evidence that GNPs–LA are able to exert both a mitochondrial protective effect on SH-SY5Y cells and a stabilizing effect on the microtubule cytoskeleton structure. Our work also demonstrated that tubulin plays a role in maintaining the cellular physiology, particularly mitochondrial functions. Indeed, the interaction between tubulin and the mitochondria is crucial in the regulation of mitochondria respiratory functions. It has been previously suggested that this interaction may involve the association of the cytoskeleton protein tubulin with the voltage-dependent anion channels of the mitochondrial outer membranes (MOM) in the control of its permeability to ADP and ATP.^73–75^ Therefore, tubulin plays a crucial role in the flux of the energy currency molecules (ADP and ATP) and so in the whole energy metabolism of the cells.

Interestingly, one of the pathological roles of the α-Syn aggregates lies on the impairment of the mitochondrial respiration Complex I that induces the selective oxidation of ATP synthase and mitochondrial lipid peroxidation.^75^ In addition, the overexpression of α-Syn induces microtubule disruption in cells,^76^ leading to a significant reduction of the acetylated tubulin level that impairs the microtubule stability. Therefore, the membrane recovery that we observed in the cytoskeleton studies when the cells were exposed to the GNPs–LA/GNPs–α-Syn mixture could be explained by the fact that GNPs–LA were able to influence the mitochondrial functions by increasing maximal respiration and ATP production and to restore the mitochondrial damage induced by the oxidative GNPs–α-Syn system.

A strong correlation between cell membrane changes and disruptions and the lipid peroxidation has been previously suggested.^61,67^

The ability of GNPs–LA to decrease both the lipid peroxidation level and the cytoskeleton damage on the cells that we observed has confirmed the correlation. Consequently, GNPs–LA are not only able to rebalance the ROS level through the restoration of the cell's antioxidant system and to regulate the mitochondrial functions, but also able to contribute to the maintenance of the microtubule network and organization.

## Experimental section

### Synthesis of gold nanoparticles (GNPs)

20 nm citrate-stabilized gold nanoparticles (GNPs) were synthesized by the sodium citrate method.^38,39^ 50 ml of 1 mM chloroauric acid solution (HAuCl_4_) was heated with stirring until boiling and different volumes of 38.8 mM sodium citrate were rapidly added. The solution was then kept at boiling for further 15 minutes to give a wine-red solution which was cooled at room temperature with stirring.

### α-Synuclein expression and labelling

α-Syn was expressed in *E. coli* using a pT7-7 plasmid. pT7-7 α-Syn WT was a gift from Hilal Lashuel laboratory (Addgene plasmid #36046). A glycine to cysteine mutation was introduced at position 7 using a Phusion Site-Directed Mutagenesis Kit (Thermo Fisher Scientific, MA, USA) for site-specific labelling. The G7C-αSyn was expressed and purified as described previously.^77^ Following α-Syn production, the G7C-αSyn was labelled with Alexa Fluor 647 C_2_ maleimide (Thermo Fisher Scientific, Massachusetts, USA) according to the instruction provided. Briefly, 10 mM dye stock solution was pre-prepared in dimethyl sulfoxide and mixed with disulphide bond reduced G7C-α-Syn solution to a final molar ratio of 3 : 1 (dye : protein). The mixture was stirred in the dark for 3 h. Then the mixture was desalted using a PD-10 desalting column containing Sephadex G-25 resin (GE Healthcare Life Sciences, Illinois, USA), and concentrated using 10k MWCO pierce protein concentrators (Thermo Fisher Scientific, Massachusetts, USA) to remove all free dye. The final labelled protein concentration was determined by UV-Vis absorbance and the labelling efficiency was determined to be 95%. The labelled samples were stored at −80 °C.

### Assembly of GNPs–LA and GNPs–α-Syn

The synthesis of GNPs–LA was performed by adaptation of the published methods.^40,41^ In brief, 1 ml bare GNP solution was diluted in 49 ml of pH adjusted Milli Q Water (pH 11, 1 M NaOH). 15 mg of α-lipoic acid (LA) (Sigma Aldrich, UK) was dissolved in 500 μl EtOH and added to the GNP solution. The reaction was left stirring overnight at room temperature in the dark. GNPs–LA were centrifuged three times at 10 000 rpm for 30 minutes at 4 °C and washed three times with pH adjusted Milli Q Water (pH 11, 1 M NaOH) to remove the excess of LA unreacted. The binding of 14 kDa α-synuclein on the surface of GNPs was carried out following the published methods^34^ using the previous expressed, purified and labelled α-synuclein. An aliquot of α-synuclein was reconstituted in HEPES buffer at 1 mg ml^−1^. 25 nM GNPs were added drop by drop to various concentrations of α-synuclein (0–5 μm) at 4 °C and incubated for 16 h to reach equilibrium. 3 μm α-synuclein was selected for the present study. The free unbound α-synuclein was then removed by three times centrifugation at 4 °C and washing with HEPES buffer. The samples were stored at 4 °C.

### Characterization of GNPs, GNPs–LA and GNPs–α-Syn

The morphology of GNPs was examined with a transmission electron microscope (Philips CM 120, TSS Microscopy, USA) using an accelerating voltage of 120 keV. UV-Vis spectra were obtained using a spectrophotometer SpectraMax M3 (Molecular Devices, San Jose, CA). The absorbance measurements were made over the wavelength range of 300–700 nm. Dynamic light scattering and zeta potential measurements were determined using a Malvern Zetasizer Nano series (Malvern Instruments, Worchestershire, UK). All the samples were analyzed at 25 °C in triplicate in both water and cell medium. AFM measurements were performed on cleaved mica substrates. 100 μl of each sample diluted in cell medium was deposited on the substrate and measured using a JPK NanoWizard 4 BioScience AFM (JPK Instruments, Germany) integrated in an iX81 optical microscope (Olympus, Belgium) operating in Quantitative Imaging mode (QI) with a silicon tip with a nominal radius of <20 nm. The images were analysed with JPK software and WSxM software.

To determine the amount of LA and α-Syn bound to GNPs, the absorption spectra of supernatants of GNPs–LA and GNPs–α-Syn were measured by UV-Vis spectroscopy. The concentrations of LA and α-Syn were calculated by their absorbance at 300 nm and 647 nm respectively. The number of LA molecules conjugated to each GNP was determined to be 20. To further confirm the presence of LA on the GNP surface, FTIR analysis was carried out on bare GNPs and GNPs–LA (Section 2.1, ESI†).

### *In Vitro* drug release

GNPs–LA were dialyzed using a Float-A-Lyzer dialysis membrane (3.5 kDa MWCO, Spectrum Laboratories, CA). GNPs–LA were then incubated with 10 mM PBS (pH = 7.4) or 10 mM acetate buffer (pH = 5.5) with agitation at 37 °C. At predetermined time points (2 h, 4 h, 6 h, 8 h, 10 h, 20 h, 40 h, 60 h, and 80 h), 1 ml of the sample was measured over *λ*_abs_ 300 nm using a SpectraMax M3 (Molecular Devices, San Jose, CA). The released medium removed was replaced with fresh buffer.

### Cell culture and viability assay

SH-SY5Y neuroblastoma cells (Sigma Aldrich UK, cat. no. 94030304) were cultured in Dulbecco's 100 modified Eagle medium (DMEM-F12, Life Technologies, UK) supplemented with 10% Fetal Bovine Serum (FBS, Life Technologies, UK) and 1% penicillin/streptomycin (P/S 100×, Life Technologies, UK). The cells were incubated at 37 °C in a humidified atmosphere of 5% CO_2_ and 95% air and confluence was achieved in about 48 h. In the cell viability assay, SH-SY5Y cells were placed in a 96-well plate. After 12 h, 48 h and 72 h exposure with the nanoparticles suspended in cell medium, the cells were washed with Dulbecco's phosphate buffer saline (DPBS, Sigma Aldrich, UK). The cells were then incubated with 100 μl per well of 3-(4,5-dimethylthiazol-2-yl)-2,5-diphenyltetrazilium bromide (MTT, VWR, UK) at 37 °C for 45 minutes. The MTT solution was removed and the cells were lysed using dimethyl sulfoxide. The samples were analysed using a PowerWave XS spectrophotometer (BioTek Instruments, UK) at *λ*_abs_ 570 nm.

### Nanoparticle uptake

SH-SY5Y cells were plated on 8-well chamber slides (Thermo Fisher Scientific, UK) at a density of 2 × 10^5^ cells per well and incubated overnight. 60 μg ml^−1^ of GNPs–LA/GNPs–α-Syn mixture in cell medium solution was added to the cells. After 1 h, 6 h, 12 h and 72 h exposure to the cells, the nanoparticles were removed, and the cells were washed three times with DPBS. 1 h prior to imaging by confocal microscopy, the nuclei of the cells were stained with Hoescht 33342 (Thermo Fisher Scientific, UK). Images were captured using a Leica SP8 inverted confocal microscope equipped with 63× and 40× objectives and processed with ImageJ software. Excitation wavelengths were 361 nm for GNPs–LA and 651 nm for GNPs–α-Syn. Emissions were collected in the 500–550 nm band region for GNPs–LA and 660–671 nm for GNPs–α-Syn. The mean fluorescence intensities were analysed using Fiji Image J software.

### Mitochondrial oxygen consumption rate (OCR) measurement

Changes in the mitochondrial respiration function were analysed using the XFe96 Analyzer from Seahorse Bioscience (Agilent Technologies, Germany). 50 000 cells per well were seeded in 24-well plates and exposed to 200 μl of GNPs (60 μg ml^−1^), GNPs–LA 1/2 (60 μg ml^−1^ and 30 μg ml^−1^), GNPs–α-Syn (60 μg ml^−1^), GNPs–LA 1/2 (60 μg ml^−1^ and 30 μg ml^−1^)/GNPs–α-Syn 1/2 (60 μg ml^−1^ and 90 μg ml^−1^), and to 6-OHDA (35 mM), for 24 h and 72 h. Before the analysis, the cells were washed with DPBS and the assay medium DMEM was added to the cells incubated at 37 °C in a humidified atmosphere of 5% CO_2_ and 95% air for 1 h. After the measurement of the baseline (three baseline measurements were recorded), the three different modulators of mitochondrial activities were added sequentially in the microplate. 1 μM oligomycin, 0.0125 μM FCCP and the 1 μM complex antimycin/rotenone were injected in succession through the ports A, B, and C of the analyser, respectively. The pH of the modulator solution was maintained at 7.4 before each experiment. For each group three independent experiments were performed. Basal respiration was calculated using the mean of the three OCR measurements before the addition of the modulators and maximal respiration was calculated after oligomycin and FCCP injection. ATP production was calculated as the portion of basal respiration that decreased after the injection of oligomycin. The spare respiratory capacity was calculated as the maximal respiration of the cells in relation to the basal respiration. OCR data were corrected for non-mitochondrial oxygen consumption after the addition of the antimycin/rotenone complex. The OCR was normalized to the cell numbers counted immediately after the completion of each experiment.

### Lipid peroxidation measurement

The detection of ROS in SH-SY5Y live cells was analysed using 2 μM BODIPY C11 581–591 (Thermo Fisher Scientific, UK). SH-SY5Y cells were plated on 8-well chamber slides (Thermo Fisher Scientific, UK) at a density of 2 × 10^5^ cells per well and incubated overnight. The lipid peroxidation was measured using confocal microscopy where the oxidized and reduced forms of the BODIPY C11 581–591 probe were excited by 488 and 561 nm, respectively. The level of lipid peroxidation was determined by monitoring the increase of the green fluorescence emission at ∼510 nm (fluorescence was measured between 500 nm and 550 nm) when the cells were exposed to 200 μl of 60 μg ml^−1^ GNPs, 60 μg ml^−1^ GNPs–LA, 60 μg ml^−1^ GNPs–α-Syn and 60 μg ml^−1^ of 1 : 1 GNPs–LA/GNPs–α-Syn mixture. Around 70 cells for each group were analysed using Fiji Image J software.

### Nano-mechanical studies

AFM studies were performed on a JPK NanoWizard 4 BioScience AFM (JPK Instruments, Germany) integrated with an iX81 optical microscope (Olympus, Belgium). SH-SY5Y cells were seeded on a glass bottomed dish and cultured at 37 °C in a humidified atmosphere of 5% CO_2_ and 95% air. The cells were exposed to 200 μl of the nanoparticle solutions (60 μg ml^−1^ of GNPs, GNPs–LA, GNPs–α-Syn and the mixture GNPs–LA/GNPs–α-Syn) in the cell growth medium for 24 h and 72 h prior to the AFM measurements. High-resolution topographical images were obtained in Quantitative Imaging mode (QI) using a silicon tip with a nominal radius of 20 nm, spring constant of 0.02 N m^−1^ and resonance frequency of 7–10 kHz. Cell elasticity measurements were performed in the Force Spectroscopy mode of JPK and spherical tips of 15 μm were used for this purpose. The data were processed by JPK software and the Young's modulus values were extracted using the Hertz model for the spherical indenter. The spring constant of the cantilever was measured before each experiment using the thermal noise method in the JPK software. All AFM studies were performed under physiological conditions at 37 °C and in the appropriate cell medium.

### Confocal imaging of the microtubule cytoskeleton

SH-SY5Y cells were plated on 8-well chamber slides (Thermo Fisher Scientific, UK) at a density of 2 × 10^5^ cells per wells and incubated overnight. 200 μl of 60 μg ml^−1^ of GNPs, GNPs–LA, GNPs–α-Syn, and GNPs–LA/GNPs–α-Syn in cell medium solutions and 6-OHDA (35 mM) were added to different wells. After 72 h exposure to the cells, the nanoparticles were removed, and the cells were washed three times with DPBS. 1 h prior to imaging by confocal microscopy, the nuclei of the cells were labelled with Hoescht 33342 (Thermo Fisher Scientific, UK) and the microtubules with SiR-tubulin (Universal Biological Ltd, UK). Images were captured using the Leica SP8 inverted confocal microscope equipped with 63× and 40× objectives. Excitation wavelengths were 361 nm for the nanoparticles and 652 nm for SiR-tubulin. Emissions were collected in the 500–550 nm band for the nanoparticles and 674 nm for SiR-tubulin. Around 70 cells for each group were analysed using Fiji Image J software.

### Statistical analysis

Data obtained from unblinded experiments are presented as mean ± standard error where the number of independent replicates was also stated. Significance of the observation was confirmed by one-way analysis of variance (ANOVA). Prism v5 (GraphPad USA) and Origin 9.0 (Microcal Software Inc., USA) were used for the statistical calculations.

## Conclusions

In summary, a drug delivery nanoconjugate system – lipoic acid capped gold nanoparticles – was developed based on the powerful natural antioxidant lipoic acid, and was then applied against the oxidative stress caused by α-synuclein aggregates in Parkinson's disease. The system, established *via* the self-assembly of the compound with the synthesized sodium citrate capped GNPs, has been shown to be bio-safe in SH-SY5Y living cells, a human neuronal cell model for the research of Parkinson's disease. The protective role of the gold nanoparticle/lipoic acid conjugate was investigated in an oxidative environment stimulated in the cells by a second drug-delivery system engineered with the same gold nanoparticle and α-synuclein.

The lipoic acid gold nanoconjugates were readily internalized and were able to restore the physiological conditions of the cells, as indicated by an increase in mitochondrial ATP production and a decrease in oxidative degradation of lipids in the cell membranes. Although further studies are required to elucidate the detailed molecular mechanism responsible for the efficacy of this nanoconjugate system, we envisage that it would be a promising treatment for the alleviation of oxidative stress in Parkinson's disease, thanks to its efficacy and biocompatibility. Furthermore, we have demonstrated that atomic force microscopy is a viable tool to gain insights into the mechanical changes of living cells due to physiological and pathology alterations. We have also shown that ROS elicit a deficit in the mitochondrial energy machinery which in turn plays a role in the microtubule structure and biophysics of the cell membrane.

However, care should be taken when delivering high concentration gold nanoparticles in a live animal study, where the residual accumulation of them in key organs might occur, a process which should be considered and monitored *in vivo*. Furthermore, new studies would be needed to fully exploit the mechanism of actions of lipoic acid since multiple mechanisms might be responsible for its neuroprotective role. Additionally, it has been reported that lipoic acid can inhibit and destabilize the formation of amyloid-β fibrils *in vitro* in a dose-dependent manner.^78^ In light of our current work and this observation, it would be desirable to evaluate if the same inhibitory effect could be observed when using the lipoic acid gold nanoparticle conjugates against the α-synuclein aggregate caused toxicity in living cell studies.

In conclusion, the present study has confirmed that the anti-Parkinson's disease lipoic acid gold nanoconjugate is promising for the treatment of oxidative stress *in vitro*. We expect that further investigations in *in vivo* experiments would bring new insights into the development of an effective antioxidant therapy for Parkinson's disease.

## Conflicts of interest

The authors declare no competing financial interest.

## Supplementary Material

NA-002-D0NA00688B-s001
