# Supplementary material for: Antioxidant lipoic acid ligand-shell gold nanoconjugates against oxidative stress caused by α-synuclein aggregates
Source: Nanoscale Adv. 2020 Oct 21;2(12):5666–81. doi: 10.1039/d0na00688b (PMC9416995; doi:10.1039/d0na00688b)
Supplement: NA-002-D0NA00688B-s001 [file NA-002-D0NA00688B-s001.pdf]

Electronic Supplementary Material (ESI) for Nanoscale Advances.
This journal is © The Royal Society of Chemistry 2020

ElECTRONIC SUPPLEMENTARY INFORMATION (ESI)

Antioxidant Lipoic Acid Ligand-Shell Gold Nanoconjugates against Oxidative Stress Caused by α-Synuclein Aggregates

Maria Elena Piersimoni[a, b], Xiangyu Teng[c], Anthony E. G. Cass[b, c] and Liming Ying[a]

[a]National Heart and Lung Institute, Imperial College London, Molecular Sciences Research Hub, London, W12 0BZ, United Kingdom.

[b]Bio Nano Consulting, London, W1T 4TQ, United Kingdom.

[c]Department of Chemistry, Imperial College London, Molecular Sciences Research Hub, London W12 0BZ, United Kingdom.

Corresponding authors: Liming Ying, E-mail: l.ying@imperial.ac.uk, Anthony E. G. Cass, E-mail: t.cass@imperial.ac.uk.

Contents

1 Supplementary Methods 3

1.1 α-synuclein Expression and Purification 3

1.2 α-synuclein Labelling 3

2 Supplementary Results 4

2.1 Nanoparticle Characterization 4

2.2 In Vitro Cytotoxicity Assay of Free Lipoic Acid and α-synuclein 5

2.3 Cellular Uptake of free LA 5

2.4 Mitochondrial Oxygen Consumption Rate (OCR) Measurement 6

2.5 Lipid Peroxidation Measurement of Free Lipoic Acid and α-synuclein 7

2.6 Nano-Mechanical Studies of SH-SY5Y Living Cells 8

.... 8

.... 9

2.7 Confocal Imaging of Microtubule Cytoskeleton of SH-SY5Y Living Cells 10

1 Supplementary Methods

1.1 α-synuclein Expression and Purification

α-synuclein was expressed using BL21(DE3) E. coli following the published method[1]. In brief, cells were grown in LB in the presence of ampicillin (100 µg/ml) at 37 °C. When the absorbance at 600 nm (OD_{600}) reached 0.5, the expression was induced by the addition of 1 mM IPTG for 3 h at 37 °C. Cells were then harvested by centrifugation in a Beckman Avanti J-26 XP centrifuge with a JLA-10.500 rotor (Beckman Coulter, California, USA) at 8000 g for 30 min. The cell pellet was resuspended in 20 mM Tris-HCl (pH 7.4), 5 mM EDTA, 1 mM protease inhibitor (cOmplete Protease Inhibitor Cocktail, Roche, Basel, Switzerland) and lysed by sonication for 2 min. Subsequently, the cell suspension was boiled at 90 °C for 20 min and then centrifuged at 16000 g with a VWR Micro Star 17 micro-centrifuge (VWR International, Pennsylvania, USA) for 20 min. The supernatant was collected and filtered with an Acrodisc 0.2 µm 25mm syringe filter (Pall Corporation, New York, USA) to remove all cell debris. After that, streptomycin sulfate was added to the supernatant to a final concentration of 10 mg/ml, and the mixture was stirred for 15 min at 4 °C. After centrifugation at 16000 g for 20 min, the supernatant was collected, and ammonium sulfate was added to 50% saturation. The mixture was stirred for 30 min at 4 °C and centrifuged again at 16000 g. The pellet was collected and resuspended in 20 mM Tris-HCl (pH 7.4), 50 mM NaCl and dialyzed overnight against 2 L of the same buffer. Protein concentration was determined from the absorbance at 280 nm with an extinction coefficient of 5960 M^{-1} cm^{-1} using a PerkinElmer Lambda 25 UV/Vis spectrometer (PerkinElmer, Massachusetts, USA).

1.2 α-synuclein Labelling

Dye-labelling of α-synuclein was achieved via a selective thiol-maleimide reaction. First, a cysteine mutation at position 7 was introduced to the wild-type protein, which is the only cysteine residue in the α-synuclein mutant that will conjugate to the Fluor 647 C_2 maleimide (Thermo Fisher Scientific, Massachusetts, USA). Prior to the reaction, 20 mM Tris-HCl (pH 7.4), 50 mM NaCl buffer was deoxygenated by bubbling with nitrogen for 1 h. Protein solution was thawed on ice, and a 10-fold molar excess of TCEP was added in protein solution to reduce disulfide bonds. Next, protein solution was eluted with the bubbled buffer through a PD-10 desalting column containing Sephadex G-25 resin (GE Healthcare Life Sciences, Illinois, USA) to remove TCEP. The concentration of eluted protein solution was determined from the absorbance at 280 nm, using an extinction coefficient of 5960 M^{-1} cm^{-1}. Subsequently, dye solution in DMSO was added to the eluted protein solution in a molar ratio of 3: 1 (dye: protein). The mixture was stirred in dark for 3 h. Then the mixture containing labelled protein was desalted and concentrated at 15000 g for 5 min, using 10K MWCO pierce protein concentrators (Thermo Fisher Scientific, MA, USA). The purified samples were stored at -80 °C. The final concentration of labelled α-synuclein was determined from the absorbance at 650 nm with an extinction coefficient of 239000 M^{-1} cm^{-1}. The total concentration of protein was

calculated using **Equation 1** below (0.03 is a correction factor for the fluorophore's contribution to the A_{280}):

$$Protein\ concentration\ (M) = \frac{A_{280} - 0.03(A_{650})}{molar\ \varepsilon\ of\ protein\ at\ 280\ nm} \quad (1)$$

From the ratio of the concentration of labelled and total protein, the labelling efficiency was determined to be 95%.

2 Supplementary Results

2.1 Nanoparticle Characterization

Table S1. Characterization of GNPs, GNPs-LA and GNPs-α-Syn by DLS (Dynamic Light Scattering) and Zeta Potential measurements. Data are represented as mean ± standard deviation, n = 3.

Type of nanoparticles	Hydrodynamic diameter (nm)	Polydispersity Index (PDI)	Zeta Potential (mV)
Bare GNPs	20	0.18	-53.6 ± 2
GNPs-LA	40	0.23	-35 ± 1
GNPs-α-Syn	60	0.20	-14 ± 2

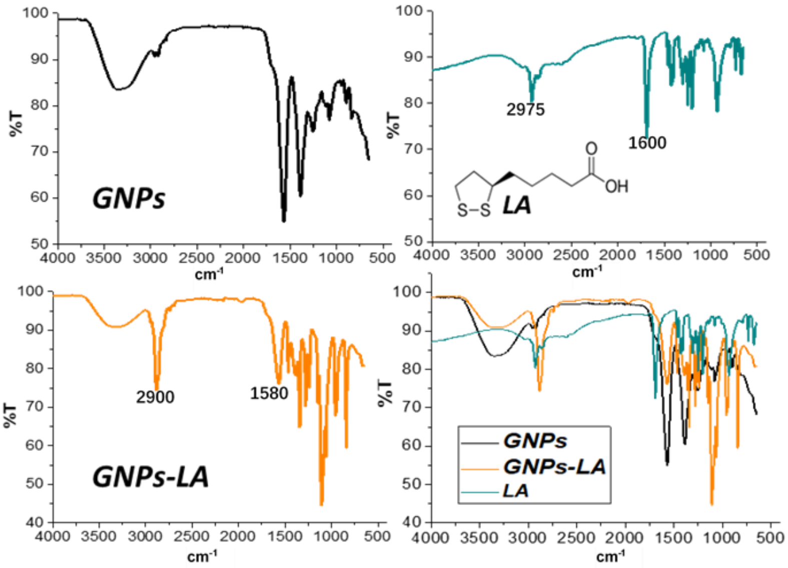

Fig S1. FTIR characterization of GNPs, LA and GNPs-LA. FTIR analysis was carried out on GNPs and GNPs-LA to further confirm the presence of the shell of lipoic acid on the nanoparticles. The spectra were analyzed in two major regions; after the coupling reaction, two main stretching that represent the footprint of lipoic acid appeared: one main peak at 2900 cm^{-1} and the other one at 1580 cm^{-1} which correspond to methylene and carbonyl stretching, respectively.

2.2 In Vitro Cytotoxicity Assay of free Lipoic Acid and α-synuclein

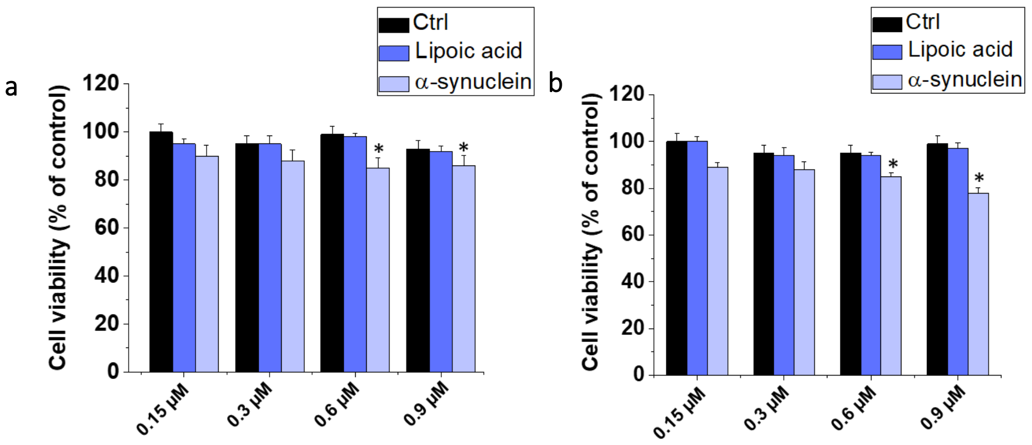

Fig S2. In vitro cytotoxicity assay of free lipoic acid (LA) and free α-synuclein. (**a**) SH-SY5Y neuroblastoma cell line was exposed to increasing concentration of LA and α-synuclein for 24 h and (**b**) 72 h. The cell viability, expressed in % in respect to the control of untreated SH-SY5Y cells (Ctrl), was assessed by MTT assay. Data are expressed as mean ± SEM, n = 3. *$P < 0.05$ in respect to the Ctrl.

2.3 Cellular Uptake of free LA

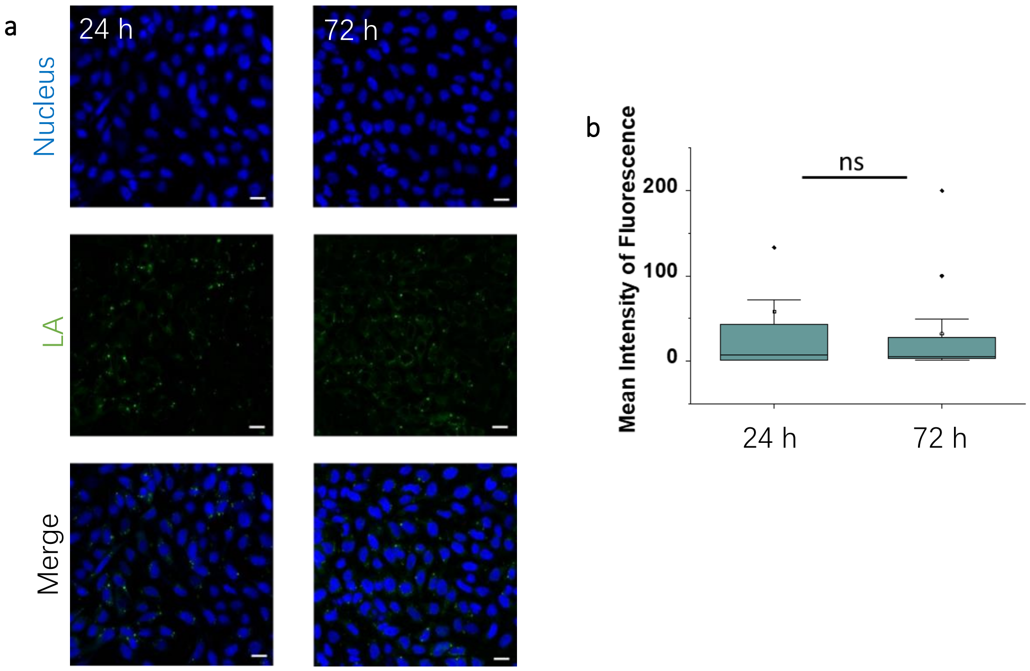

Fig S3. Intracellular uptake and distribution of 0.6 µM free LA. (**a**) Live SH-SY5Y cells were exposed to LA 24 h and 72 h prior to imaging by confocal microscopy. The nucleus is visualized in blue and lipoic acid in green (AlexaFluor 647 label). Scale bar = 10 µm. (**b**) The mean fluorescence intensity of LA by confocal microscopy in SH-SY5Y cells after 24 h and 72 h LA exposure. Data are given as mean ± SEM; n=3. Ns, not significant.

2.4 Mitochondrial Oxygen Consumption Rate (OCR) Measurement

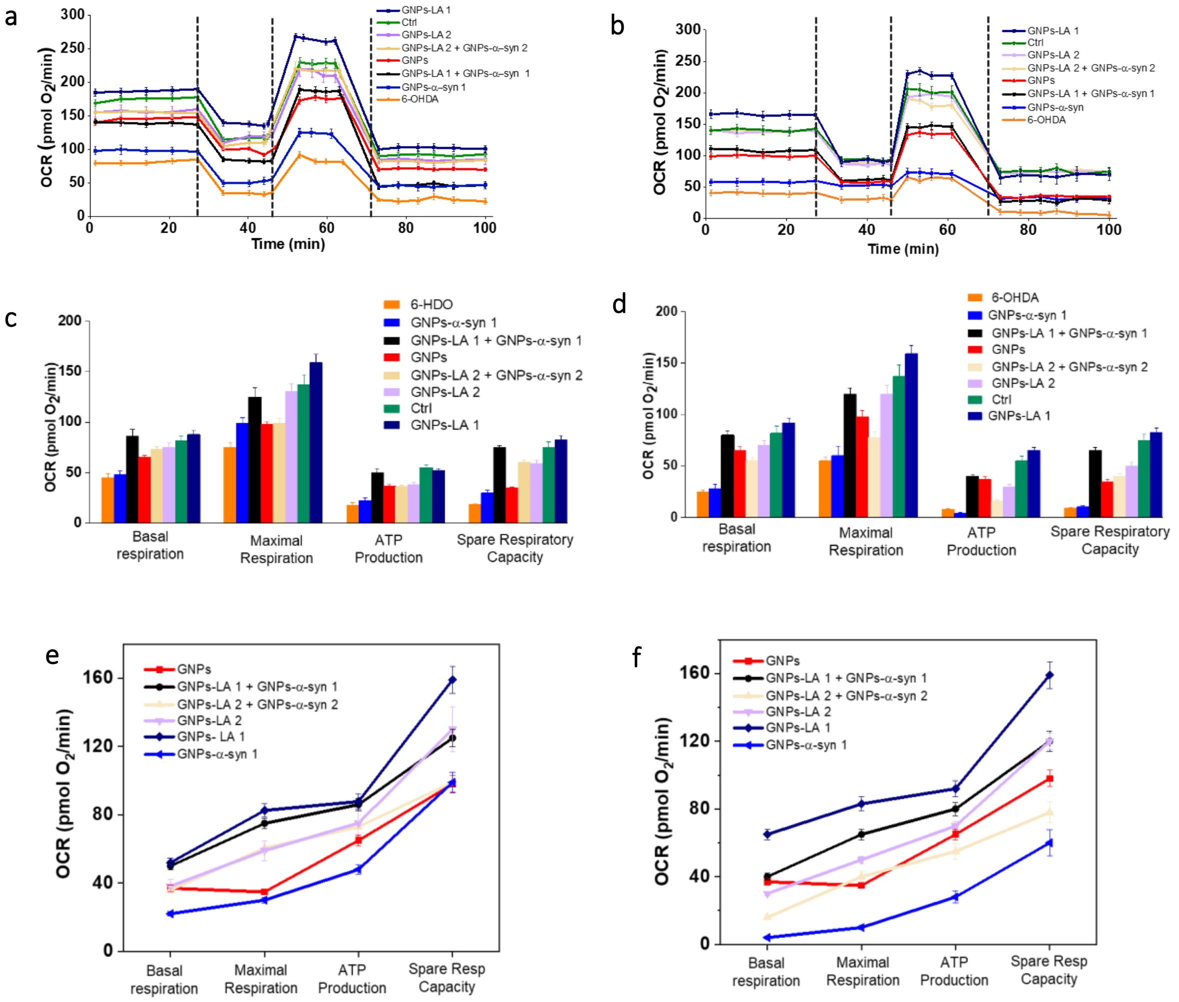

Fig S4. Mitochondrial oxygen consumption rate (OCR) measurement by SeahorseXFe96 in SH-SY5Y live cells. (**a-b**) OCR throughout mitochondrial function testing of SH-SY5Y after 24 h and 72 h exposure to either vehicle (Ctrl), GNPs-LA 1 (60 µg/ml) and 2 (30 µg/ml) , GNPs-α-Syn (60 µg/ml), GNPs (60 µg/ml), GNPs-LA 1-2 (60 and 30 µg/ml)/GNPs-α-Syn 1-2 (60 and 90 µg/ml) mixture and 6-hydroxydopamine (6-OHDA). (**c-d**) Basal Respiration, Maximal Respiration, ATP Production and Spare Respiratory Capacity were calculated from OCR of SH-SY5Y live cells exposed for 24 h and 72 h to either vehicle (Ctrl), GNPs-LA 1 (60 µg/ml) and 2 (30 µg/ml) , GNPs-α-Syn (60 µg/ml), GNPs (60 µg/ml), GNPs-LA 1-2 (60 and 30 µg/ml)/GNPs-α-Syn 1-2 (60 and 90 µg/ml) mixture and 6-hydroxydopamine (6-OHDA). Values are reported as mean ± SEM (n=12 per group). (**e-f**) Effect of 24 h and 72 h exposure of GNPs (60 µg/ml), GNPs-LA 1 (60 µg/ml) and 2 (30 µg/ml), GNPs-α-Syn (60 µg/ml), GNPs-LA 1-2/GNPs-α-Syn 1-2 on mitochondrial function parameters in SH-SY5Y living cells.

2.5 Lipid Peroxidation Measurement of Free Lipoic Acid and α-synuclein

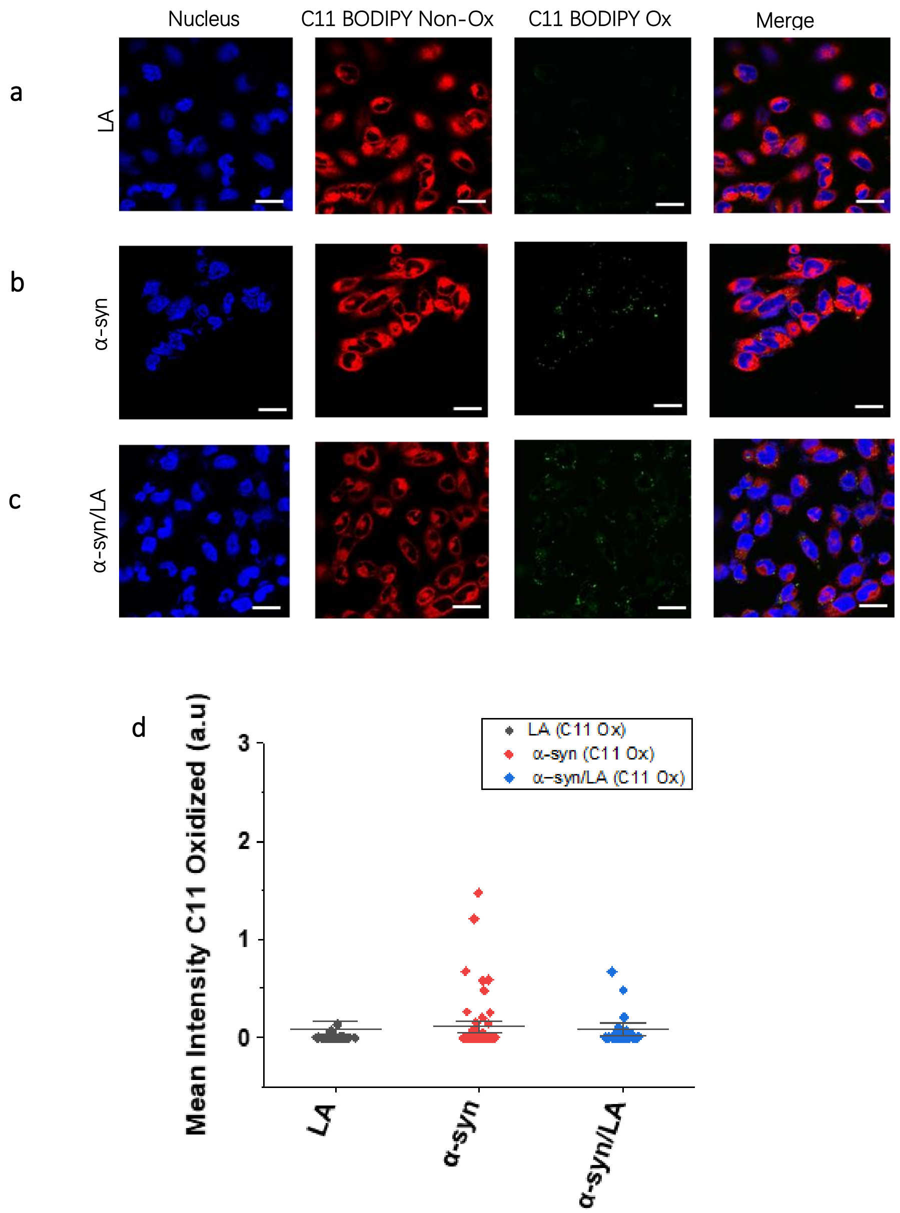

Fig S5. Lipid peroxidation measurements using C11 BODIPY (shift of fluorescent emission peak from 590 nm red to 510 nm green). (**a**) Lipid peroxidation levels after SH-SY5Y cells exposure to 0.6 µM of LA and (**b**) 0.6 µM α-Syn and (**c**) 0.3 µM each α-Syn/LA together prior to imaging by confocal microscopy. The nucleus is visualized in blue. Scale bar = 10 µm. (**d**) The mean fluorescence intensity of the shift of the emission peak from non-oxidized (red) to oxidized (green) for each experiment. Data are given as mean ± SEM; n=3.

2.6 Nano-Mechanical Studies of SH-SY5Y Living Cells

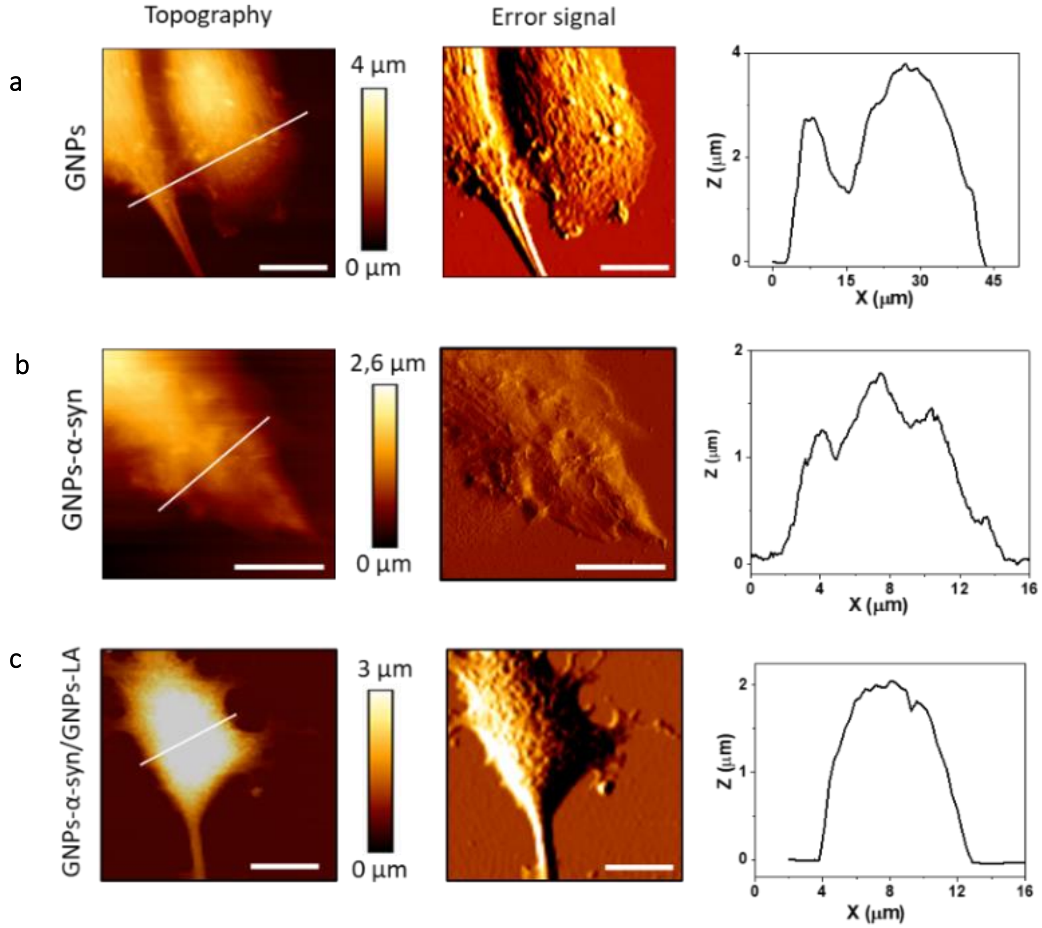

Fig S6: (**a**) Topographic AFM images and error images of SH-SY5Y living cells exposed to 60 µg/ml GNPs, (**b**) 60 µg/ml GNPs-LA and (**c**) 60 µg/ml of GNPs-α-Syn (30 µg/ml) /GNPs-LA (30 µg/ml) mixture. Corresponding cells height profile across the lines in the topographic AFM images on the right panel. Scale bar = 10 um.

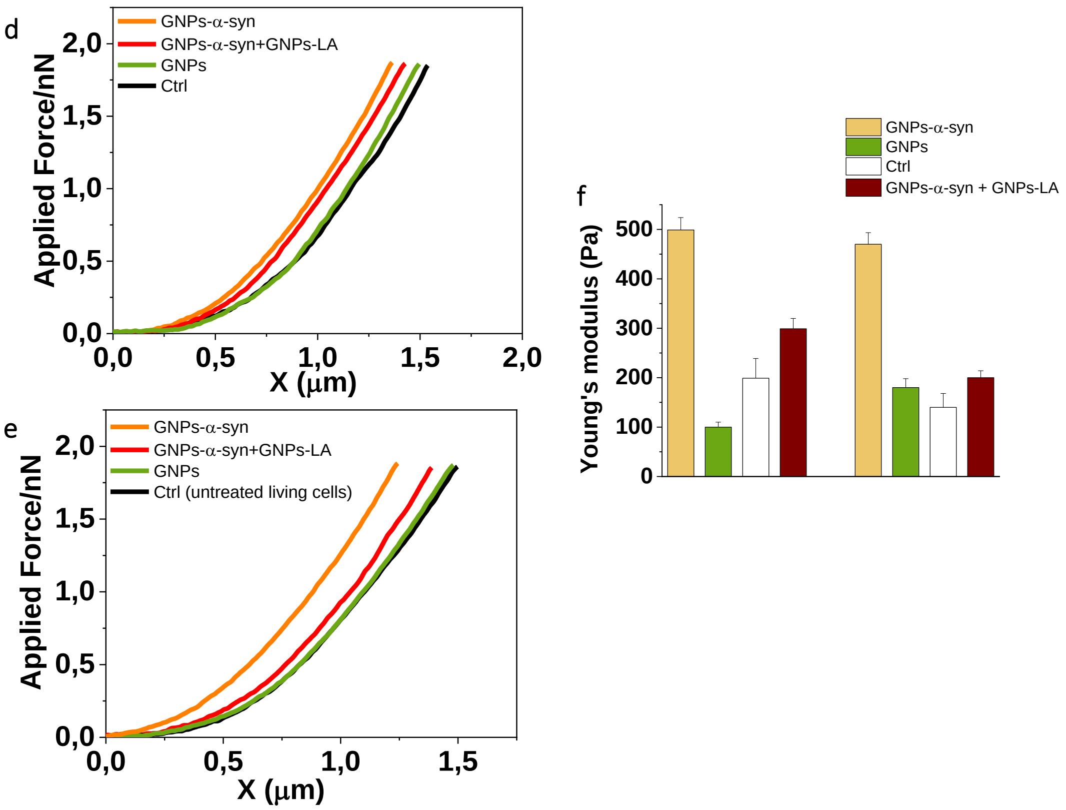

Fig S7. (**d-e**) Examples of force-indentation experimental curves recorded for both untreated (Ctrl) and treated SH-SY5Y live cells. SH-SY5Y live cells were exposed to 60 µg/ml GNPs , 60 µg/ml GNPs-α-Syn and 60 µg/ml of GNPs-LA/GNPs-α-Syn (30 µg/ml each) mixture for 24 h and 72 h. (**f**) Young's modulus mean value obtained from AFM measurements on SH-SY5Y living cells after the exposure to GNPs-α-Syn, GNPs and the mixture GNPs-α-Syn/GNPs-LA for 24 h (left) and 72 h (right). Error bars represent the standard error of the mean for each condition.

2.7 Confocal Imaging of Microtubule Cytoskeleton of SH-SY5Y Living Cells

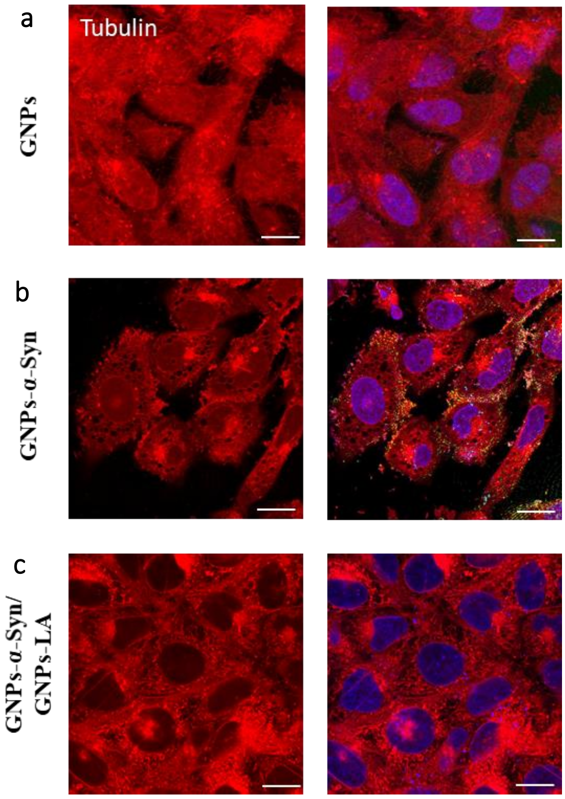

Fig S8. Changes in the microtubule cytoskeleton induced by 72 h exposure to the nanoparticles investigated by confocal imaging using SiR-Tubulin protein. (**a**) SH-SY5Y cells treated with 60µg/ml GNPs. (**b**) and (**c**) are SH-SY5Y treated with 60 µg/ml GNPs-α-Syn and 60 µg/ml GNPs-α-Syn/GNPs-LA (30 µg/ml each) mixture. Scale bar = 10 µm.

Reference

1. W. Hoyer, T. Antony, D. Cherny, G. Heim, T. Jovin, V. Subramaniam, Dependence of α-Synuclein aggregate Morphology on Solution Conditions, *J. Mol. Biol.*, 2002, 322, 383–393.
